# Taking mental health into the community

**DOI:** 10.2471/BLT.14.021014

**Published:** 2014-10-01

## Abstract

The Czech Republic’s plan to pilot community-based mental health services is a welcome development for people with both mild and severe forms of mental illness. Petr Třešňák reports.

For 34-year-old Jana Stastny (not her real name) things started to fall apart when she broke her leg. She was still living with her husband at that time, sharing an apartment in the Czech Republic capital, Prague. Confined to her bed, Stastny started to suffer delusions.

“I had this obsessive feeling that there was a war outside, and that I could easily become a victim,” she says. After several months of suffering, and fearing for her sanity, Stastny called the emergency services and she was admitted to a psychiatric hospital in the city, where she was initially put in a ward with severely agitated patients.

“It was hell on earth,” she remembers. She was diagnosed with bipolar disorder, and released after a few months.

When she returned home her husband was living with another woman. “I tried to kill myself and ended up being re-admitted,” she says. Her second stay in hospital ended when she managed to escape during an afternoon walk. Back on the outside, and without support or guidance, she soon fell apart again. A pattern was being established: periods of terrifying institutional care followed by periods of terrifying struggle outside.

Everything changed, however, when Stastny moved to Prague’s District 8 and was admitted to the Bohnice Psychiatric Hospital. Run by Dr Martin Holly, the Bohnice is the largest psychiatric facility in the country. 

It has some 1200 beds, half of which are occupied by elderly psychiatric patients and substance abuse patients, while the rest are divided between long- and short-term patients living with a range of disorders (around 200 and 400 patients respectively). Attached to the hospital is the Bohnice Hospital Community Centre, which offers care and support for about 100 patients, including Stastny, who are living at home.

**Figure:**
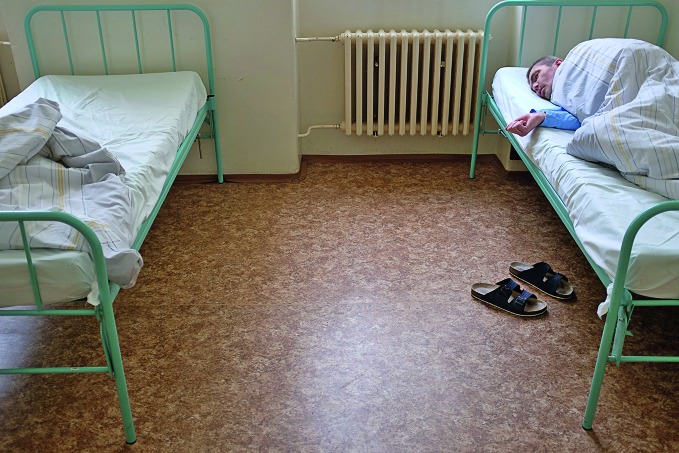
A patient sleeping in Ward 14 of the Bohnice Psychiatric Hospital in Prague

Martin Svozílek

Stastny noticed two big differences between the care offered at the Bohnice and the care she received before: first, she was treated as an individual; second, from the outset, the Bohnice focused on getting her home to live as independently as possible in the community.

“I had visits not just from nurses but also social workers,” she says. “They talked to my doctor about my illness, but they also asked me how I felt and what I needed for a successful return home.” 

Then, once she was back home, the support continued, with nurses helping her to readjust to everyday life at home and deal with administrative matters, such as claiming disability allowance so that she could pay her bills. “Without that support I would have quickly ended up homeless again,” she says.

The civil rights movement of the 1960s and 1970s brought reforms in some countries of the mental health care systems that started to respect individual rights and encourage patients to live at home, supported by health and social workers. Such reforms have got under way in some east European countries, including the Czech Republic following the Velvet Revolution in 1989. 

“This is a welcome development and very much in tune with the World Health Organization’s (WHO) approach to mental health,” says Dr Michelle Funk, coordinator of the Mental Health Policy and Service Development unit at WHO headquarters in Geneva. “The idea is to move from a medical model focused on symptom reduction to one that focuses on recovery, thus supporting people to live a fulfilling life in society.”

“The idea is to move from a medical model focused on symptom reduction to one that focuses on recovery, thus supporting people to live a fulfilling life in society.”

Michelle Funk

The Czech mental health system came under criticism in recent years for its focus on large asylums and mistreatment of patients including their confinement in so-called “cage beds”. Despite this criticism, efforts to reform the system have been slow.

To support such countries, WHO launched a new campaign in 2013, called QualityRights. This month the organization is devoting its annual campaign on World Mental Health Day to schizophrenic disorders, stressing that even people with these and other severe mental illnesses can live in the community given the appropriate care and support.

“WHO is working to end the institutionalization of people with mental health problems, since institutions are associated with human rights violations, and to set standards for the delivery of inpatient and outpatient services that promote human rights and independent living in the community,” Funk says.

One of the main reasons for slow progress in the Czech Republic has been the lack of consultation between the main stakeholders, according to Dr Ivan Duškov, head of the Strategic Project Management Office at the Ministry of Health. To improve the dialogue between key players in the mental health system, a working group representing community services, the ministry of labour and social affairs, health insurers, patients and carers, was set up in 2012.

The group spent a year hammering out a reform strategy based on what is known as a balanced care model, in which psychiatric hospitals continue to offer care for the severest cases, while people living with less severe forms of mental illness are cared for by a network of community establishments called mental health centres.

“The mental health centres are the big news,” says Duškov, who hopes that these will not only increase the number of facilities offering care but also provide mobile teams to support people in their homes. The aim is to offer the kind of care already provided by the Bohnice.

Martin Holly is an enthusiastic supporter of reform, and is watching developments closely. So far, no decision has been taken regarding the exact form the mental health centres will take. “The plan is to start with about 20 pilot mental health centres to find the best working model,” he says. “This is not just about identifying the specifics in different settings, but, for example, finding out who will be the best service provider in the mental health centres. Should it be a big hospital, a nongovernmental organization with relevant experience, or a completely new entity? We have to find out.”

**Figure:**
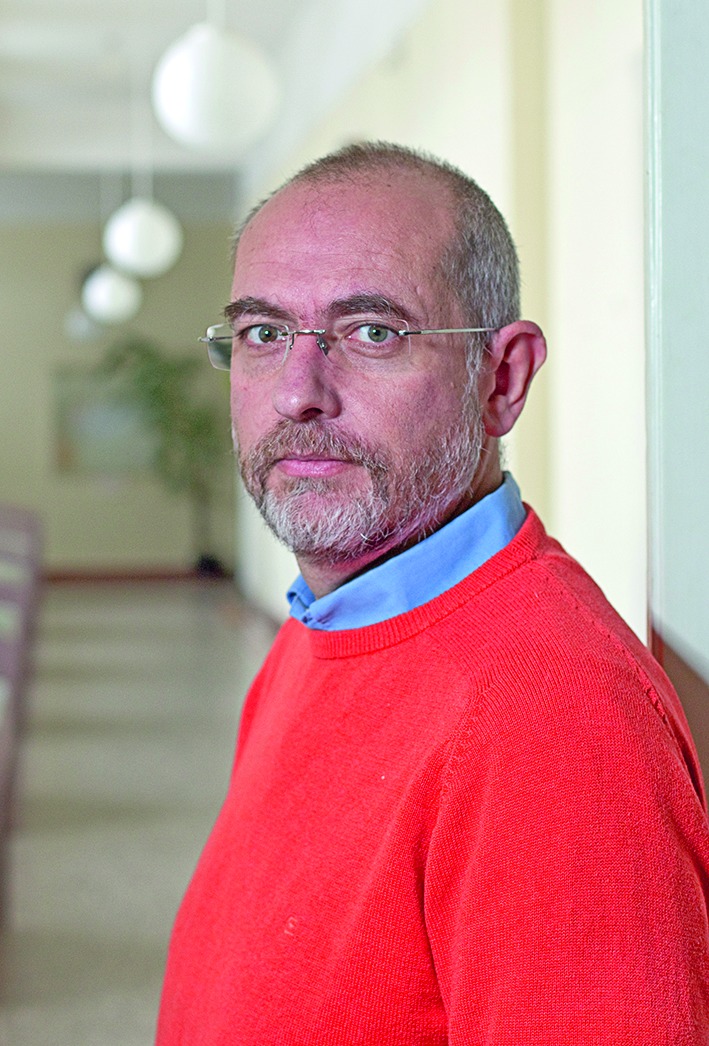
Dr Martin Holly, director of the Bohnice Psychiatric Hospital

Martin Svozílek

Policy-makers will also have to find out how much it will all cost. Providing the kind of intensive personal care offered by the Bohnice could be expensive.

According to Duškov, the Czech government will be tapping into European Union funding to finance the reform, but stresses that these funds would be to facilitate reform rather than fund a new system. “When the reform process is finished, the system will be financed solely from our local sources,” he says, underlining the importance of financial sustainability.

Holly believes however that reform may actually save money, if the Bohnice is any sort of guide. “We care for about 100 patients living in the community and spend around 5 million Czech koruna (US$ 251 000) per year on this, which is the cost of 10 hospital beds,” he says, adding that the continuous fieldwork of the nurses and doctors helps to avoid hospitalizations, saving more beds, around 15 to 20 a year, and even more money.

Holly envisages centres serving between 100 and 200 patients, which would allow for personal relationships to develop, a crucial aspect of quality mental health-care provision. He is also keen to see mental health centres that offer care 24 hours a day.

While optimistic about the prospects for the balanced care approach, Holly believes that it will be vital to continue support and funding for some parts of institutional care to cover the needs of people living with the severest forms of mental illness. While WHO recommends residential housing for such patients, Holly explains that it is not current government policy to provide this and that additional funds would be needed. At the Bohnice, care for the most challenging and challenged patients is the responsibility of Ward 14, which is run by head carer Jan Uhlíř.

Like Holly, Uhlíř believes that some patients can only be cared for in the ward. “The majority of our patients have been hospitalized almost continually,” he says. “We think that for some of them there is no other way as they lack the basic ability to take care of themselves. There are patients who could put themselves at risk or people around them.”

Uhlíř does believe, however, that increased funding for mental health care could allow for a more ‘normal life’ to come into the wards. “I would like to make life here as similar to the life outside as possible. If we had 10 patients less, we could remove two beds from each room and put in an armchair and some cabinets for patients to keep their personal belongings instead.”

Despite his clear-eyed view of the challenges many of his patients face, Uhlíř is a great believer in the healing effect of socialization. “In my experience, if the patient is given appropriate care, if there is a proper re-socialization programme, even a patient with severe mental illness can enjoy a certain degree of self-reliance in a residential care home,” he says.

“If you just give patients a piece of paper saying that they are discharged from the hospital, the moment something goes wrong, everything starts to fall apart … and, pretty soon, they are back here.”

Jan Uhlíř

For those who are able to return to normal life, Uhlíř stresses the importance of continued support. “If you just give patients a piece of paper saying that they are discharged from the hospital, the moment something goes wrong, everything starts to fall apart. Then they fall apart and, pretty soon, they are back here,” he says.

Stastny knows what it feels like for her life to fall apart, and to be saved. “If I get worse, I’m not afraid to go back to hospital anymore,” she says “because I know that the community nurses will help me recover.”

